# Supplementary material for: Chest drain REgular FLushing in ComplIcated parapneumonic EFfusions and empyemas: Study protocol for the RELIEF randomized controlled trial
Source: PLoS One. 2026 Mar 5;21(3):e0331725. doi: 10.1371/journal.pone.0331725 (PMC12962536; doi:10.1371/journal.pone.0331725)
Supplement: S1 File — (DOCX) [file pone.0331725.s001.docx]

**Chest Drain Regular Flushing in Complicated Parapneumonic Effusions and Empyemas**

**Version.02**

**(RELIEF Study)**

**Principal Investigator**

**Samira Shojaee, MD, MPH**

Contents

[**1.** **List of Abbreviations** 1](#_Toc147829934)

[**2.** **General Study Information** 1](#_Toc147829935)

[**3.** **Objectives** 1](#_Toc147829936)

[**4.** **Rationale** 1](#_Toc147829937)

[**4.1.** **Background** 1](#_Toc147829938)

[**4.2.** **Hypothesis** 3](#_Toc147829939)

[**5.** **Design** 3](#_Toc147829940)

[**5.1.** **Study Population** 3](#_Toc147829941)

[**5.2.** **Study Criteria** 3](#_Toc147829942)

[**5.3.** **Definitions** 4](#_Toc147829943)

[**5.4.** **Target Accrual** 4](#_Toc147829944)

[**5.5.** **Enrollment** 4](#_Toc147829945)

[**6.** **Research Plan and Method** 5](#_Toc147829946)

[**6.1.** **Study Procedure** 5](#_Toc147829947)

[**6.2.** **Outcomes** 6](#_Toc147829948)

[**6.3.** **Design** 6](#_Toc147829949)

[**6.4.** **Data Collection** 6](#_Toc147829950)

[**6.5.** **Multistate Transition Model:** 8](#_Toc147829951)

[**7.** **Statistical Considerations** 9](#_Toc147829952)

[**8.** **Informed Consent/Authorization** 10](#_Toc147829953)

[**9.** **Data Confidentiality and Monitoring** 11](#_Toc147829956)

[**10.** **Safety** 11](#_Toc147829957)

[**11.** **Finances** 12](#_Toc147829958)

[**12.** **References** 14](#_Toc147829959)

# **List of Abbreviations**

| VUMC | Vanderbilt University Medical Center |
| --- | --- |
| CPPE | Complicated parapneumonic pleural effusion |
| RCTs | Randomized controlled trials |
| BTS | British Thoracic Society |
| tPA/DNase | Intrapleural tissue plasminogen activator and dornase alfa |
| LDH | Lactate dehydrogenase |
| VATS | Video-assisted thoracoscopic surgery |
| CRF | Case report form |
| REDCap | Research Electronic Data Capture |
| DAGs | Data Access Groups |
| AE | Adverse event |
| SAE | Serious adverse event |

# **General Study Information**

**Principal Investigator**: Samira Shojaee, MD, MPH

**Biostatistician**: Heidi Chen, PhD

**Co-investigators:** Jennifer Duke, MD, Fabien Maldonado, MD, MSc, Robert Lentz, MD,

Vanderbilt University Medical Center

**Title**: Chest Drain **Re**gular F**l**ushing in Compl**i**cated Parapneumonic **Ef**fusions and Empyemas

(RELIEF Study)

# **Objectives**

The primary objective of this study is to evaluate the impact of regular chest tube flushing on the length of time to catheter removal for patients admitted with an infected pleural space who require thoracostomy for the management of a complicated parapneumonic pleural effusion (CPPE) and/or empyema.

The secondary objective of this study is to evaluate the impact of regular chest tube flushing on the length of total hospitalization for patients, radiographic improvement on chest imaging, the need for additional pleural interventions, and associated complications of chest tube management. We will employ ordinal multi-state transition modeling to analyze the magnitude of the intervention’s impact on successful or failed outcomes over the course of the chest tube dwell period.

# **Rationale**

There are no randomized controlled trials (RCTs) evaluating the role of regular chest tube flushing in the setting of pleural space infection for optimal drainage and treatment outcomes. Most studies of <16 Fr catheters have used both flushing and suction to decrease the likelihood of catheter blockage and improve drainage efficiency, however, this practice has never been studied prospectively or in RCTs. Regular flushing (e.g., 20-30 ml saline every 6 h via a three-way stopcock) is recommended for small chest drains by the British Thoracic Society (BTS) 2010 Guidelines and not addressed in the most recent 2023 guidelines. Regular flushing is followed variably by some and not used by others. Importantly, the role of this practice in successful drainage of infected fluid, and patient-centric outcomes has not been investigated. Inconsistent flushing practices confound the interpretation of therapeutic modalities (such as tPA/DNase) success or lack thereof and limit the execution of RCTs and prospective studies of the pleural space in the setting of infection.

# **Background**

Pleural effusions are a frequent complication of acute pulmonary infections and can impact patient morbidity and mortality (Sorino). For example, of those hospitalized with pneumonia, 20% to 40% have a parapneumonic effusion, and 5% to 10% of these parapneumonic effusions progress to empyema with approximately one-third of patients requiring surgical drainage of the pleural space (Najafi, Shen). Based on the 2016 National Hospital Care Survey, nearly 77,000 adult patients were hospitalized for pneumonia with 35% of inpatients dying either in the hospital or within 1 year of discharge (Peters). Those with pleural space infections often require additional procedures with increased length of hospital stay.

The three stages of parapneumonic pleural effusion include (1) simple exudative accumulation (2) fibropurulent with bacterial invasion of the pleural cavity, and finally, (3) the organized stage with the formation of scar tissue (Sorino). A parapneumonic effusion in the fibropurulent stage is usually defined as “complicated” since antibiotic therapy alone is not sufficient for its resolution and an invasive procedure (pleural drainage or surgery) is required. Biochemical features of a fibrinopurulent collection include a low pH (<7.2), low glucose level (<60 mg/dl), and high lactate dehydrogenase (LDH). Empyemas are often defined as frank pus aspirated from the pleural space, with some further defining positive gram stain and/or culture positivity as empyemas (Shen, Light, Davies).

The most recent BTS guidelines recommend that patients with frankly purulent, turbid/cloudy pleural fluid, or with a pH <7.2 on sampling should receive prompt pleural space chest tube drainage, although there is no consensus on optimal thoracostomy size in the absence of a randomized controlled trial (Davies). In a subanalysis of the Multicenter Intrapleural Sepsis Trial (MIST1) cohort, there was no increased efficacy with large-bore tubes compared with small-bore drains (Maskell).

One criticism of the use of smaller bore chest tubes for pleural infection management is the risk of drain obstruction which could be ameliorated through flushing of the catheter with sterile saline. The BTS guidelines recommend regular flushing (20–30 ml saline every 6 h via a three-way tap) for smallbore catheters and the application of suction (−20 cm H2O) in the hopes of improved drainage (Davies). However, there is no RCT evaluating these drain management issues, including flushing and drain suction.

In the observational retrospective study by Cafarotti et al., ninety-seven 12F drains were placed for empyemas with 62.8% of drains were ultimately removed secondary to blockage, often with observed fibrin or blood in the drain. Drain flushing was performed with 50 mL of saline solution in case blockage was suspected (Cafarotti). Similarly, a retrospective review of one hundred 12F chest tubes (20% placed for pleural infections) found that 9% of the drains became blocked (Davies). However, 58 catheters were flushed regularly with 20 ml of sterile saline every 6 hours, and found that the frequency of drain blockage in pleural effusion was reduced by administration of regular normal saline drain flushes (odds ratio for blockage in flushed drains compared with non-flushed drains 0.04, 95% CI: 0.01–0.37, p<0.001) (Davies). Additionally, there appears to be support for the addition of saline flushing when administering intrapleural lytic therapy with less overall doses of fibrinolytics needed in the group of patients receiving saline (and no adverse events attributed to saline therapy) (Porcel).

These findings are not consistent across all studies. Horsley et al. prospectively analyzed 52 catheters (ranging from size 12-20F) and found that 6 of the 10 drains placed for empyema management were complicated by blockage. Regular flushing with 30 mL saline four times daily was used in the clogged drains and in an additional five chest tubes that did not block. Flushing drains with saline solution did not appear to alter the chance of blockage (Horsley). Many centers within and outside the US, do not follow regular flushing protocols and instead, evaluate for blockage and only flush if a catheter appears blocked and non-draining. This is further supported by studies of pleural space infection that do not report a chest tube flush protocol. As a result, the speculation of “flush role in the outcome and successful chest tube removal” is the standard study limitation listed in most pleural space infection studies.

This inconsistency in prior literature and the absence of RCTs for optimal drain management has led to variability in practice despite the BTS 2010 guidelines. We propose a multicenter trial evaluating the utility of regular chest drain flushing of catheters placed for the management of pleural space infection.

# **Hypothesis**

Regular thoracostomy flushing of catheters placed for the management of pleural space infection leads to earlier successful chest tube removal. This, in turn, may lead to improved overall outcome measures such as earlier discharge from the hospital, optimal resolution of pleural space infection, prevention of surgical management, or additional invasive procedures in the pleural space (such as additional drains).

# **Design**

This study will be a multicenter, open label randomized controlled trial.

# **Study Population**

All consecutive patients with a pleural space infection requiring chest tube placement for inpatient management will be screened for study inclusion.

# **Study Criteria**

**Inclusion Criteria**

- Patients with CPPE and empyema requiring chest tube placement as standard of care for inpatient management of their pleural space infection with or without intrapleural tPA/DNase therapy
- Age > 18 years old.

**Exclusion Criteria**

• Chest tube has been in situ >24 hours.

• Patients who have chest tubes that can’t accommodate a three-way stopcock.

• Chest tube is an indwelling tunneled pleural catheter.

• Study subject has any disease or condition that interferes with the safe completion of the

study.

• Inability to provide informed consent.

• If the managing clinician believes the chest tube will be placed for less than 24 hours.
• Patients admitted with an indwelling chest tube following pleuroscopy or alternate pleural procedure preceding tube placement.

• Patient has already undergone a pleural procedure for the treatment of the pleural infection of interest (ex: prior chest tube placement or VATS with decortication).

# **Definitions**

A *complicated parapneumonic pleural effusion (CPPE)* is an effusion that has been infected with bacteria or other microorganisms (e.g., positive Gram stain or culture) or biochemical evidence of marked inflammation (e.g., low pH (<7.20), low glucose level (<60 mg/dl), and high lactate dehydrogenase (LDH)).

An *empyema* is a pleural effusion in which frank pus is aspirated from the pleural space.

A *blocked chest tube* is a chest tube that has stopped draining pleural fluid due to particulate material in the lumen of the tube.

A *successful chest tube removal* occurs when the infection in the pleural space has resolved as defined by clinical improvement of the patient and 24-hour fluid output from the chest tube of 150 mL or less.

# **Target Accrual**

We plan to enroll a total of 96 patients who will be randomized with a 1:1 ratio to the interventions (regular saline flushing) or the control (standard of care) arms. We will generate the randomization sequence using a computer program.

# **Enrollment**

The screening and enrollment of subjects will be done by a study coordinator or an investigator who is a member of the RELIEF research team within Vanderbilt or participating institutions. The study coordinator will be responsible for ensuring and reporting subject screening for study eligibility. Once the investigator has determined the subject’s eligibility for the study, the background of the proposed study and the benefits and risks of the study and procedures will be explained to the subject as a part of the consenting process. After consenting, subjects will be considered enrolled if they meet all the inclusion criteria and none of the exclusion criteria. Subjects who fail to meet any of the entry criteria will be excluded from the study and considered a screen failure. Screen failures will be recorded without patient information. Subjects will be able to withdraw from the study at any point.

# **Research Plan and Method**

This will be a randomized trial of patients with chest tubes placed per standard of care for the management of pleural space infection.

The primary objective of this study is to evaluate the impact of regular chest tube flushing on the length of time to catheter removal for patients admitted with pleural space infection who require thoracostomy for the management and clearance of infected pleural fluid.

The secondary objective of this study is to evaluate the impact of regular chest tube flushing on the length of hospitalization for patients, radiographic improvement on chest imaging, the need for additional pleural interventions, and associated complications of chest tube management. We will employ multi-state transition modeling to characterize the role of flushing in longitudinal clinical outcomes and to scrutinize the role of flushing between the two arms in a more granular fashion.

# **Study Procedure**

Patients will be eligible for randomization immediately following and up to 24 hours after chest tube placement. As per the standard of care, patients with a chest tube require a chest X-ray after chest tube placement to assess the proper position of the chest tube. Chest X-ray and ultrasound images obtained at the time of chest tube placement will be reviewed and their information documented as baseline/ day 1. The study team will obtain a baseline assessment of the pleural effusion via ultrasound if no ultrasound images are available within 24 hours prior to chest tube placement).

Subjects will be randomly allocated into intervention (regular flushing) and control (standard of care/flush as needed) groups using computer-generated randomization just prior to starting the procedure.

The intervention groups will have sterile saline 20 mL flushed into their catheter by trained nurses or study team members every 6 ± 2 hours. If patients are receiving tPA/DNase (combined at the same time), each treatment will be considered one flush.

The control groups will have no saline routinely instilled into the chest tube. Instead, chest tube patency will be assessed at least once/day. If the chest tube is considered blocked (lack of tidaling, lack of fluid drainage, and presence of fluid on US exam), the chest tube will be flushed with 20 mL of saline if indicated. All patients in both arms will have -20 cm H2O at the atrium (-80 cm H2O at the wall) continuous suction applied to their drainage system at all times if tolerated.

A daily checklist will be completed by the research team to assess for chest tube blockages and ongoing need for the tube/potential removal of the catheter (see CRF).

On the day of chest tube removal, both the intervention group and control group will undergo a repeat chest X-ray. Post-chest tube removal ultrasound images will be obtained by the research team. Chest X-rays can be performed as portable semi-erect or PA and lateral based on the clinical judgment of the primary provider.

# **Outcomes**

Primary outcome: time from randomization (within 24 hours of chest tube placement) until time to chest tube removal (hours).

Secondary outcomes:

1 – length of hospitalization.

2 – radiographic improvement as evidenced by chest x-ray at the time of chest tube placement compared to the time of removal.

3 – additional surgical procedures for the management of pleural space infection (VATS, additional drains, etc.).

4 – Complications.

# **Design**

Prospective multicenter randomized controlled trial, open label.

# **Data Collection**

The following data will be collected from the records:

1) Demographic data – age, gender, race, smoking status

2) Indication for chest tube placement

3) Date and time of catheter placement

4) Amount of daily fluid drained while in place

5) Imaging data (Chest x-ray and ultrasound images)

6) Length of hospitalization

7) Any instances of deviations from an assigned group (e.g. if a drain was thought clogged and

underwent saline flushing outside of the protocol)

8) Replacement of catheter secondary to dislodgement or inability to drain

9) Presence or absence of continuous wall suction application (for >75% of the previous 24 hours)

10) Date and time of catheter removal

11) Data related to daily chest tube blockage assessment and chest tube removal objective data.

(Because the study investigators and participants are not blinded to the study arm, specific objective criteria (see CRF) for chest tube removal due to successful pleural space infection management will be assessed on daily bases. These data will be documented to assess for change in the behavior of provider due to lack of blinding. This includes categorization of daily drain status, meeting either “successful,” “ongoing” or “”failed criteria.” This data will be used to perform sensitivity analysis to address the potential effects of blinding. Research team members will capture this data.

13) Imaging data on day of chest drain removal (ultrasound, chest-X-ray, or CT scan) characterizing effusion status (Table 2).

**Table 2. Imaging data collected on day of chest drain removal**

| **Day of Chest Drain Removal: Effusion Classification** |
| --- |
| **Chest X-Ray** |
| 0= no pleural fluid present  1= blunting of the costophrenic angle  2= fluid occupying up to 25% of the hemithorax  3= fluid occupying between 26-50% of the hemithorax  4= fluid occupying between 51-75% of the hemithorax  5= fluid occupying between 76-100% of the hemithorax |
| **Ultrasound** |
| Number of rib spaces occupied by effusion in the:  -Anterior view (#)  -Axillary view (#)  -Posterior view (#) |
| **CT Scan** |
| Effusion characteristics:  -Presence or absence of loculations  Effusion size in non-loculated effusion:  - Scant volume/small  - ≤ 1/4 of hemithorax  - ¼ to ≤½ of hemithorax  - ½ to ≤ 3/4 of hemithorax  - ¾ to almost entire hemithorax |

12) Follow up-Data

1. Other treatment strategies: including presence of additional chest tube in the same pleural space, surgical consult (and if applicable surgery date and interventions).
2. Post hospitalization data: Including date of discharge, status of empyema 4-6 weeks and 3 months after removal, and date of infection resolution on chest imaging
3. Complications: Including procedural and treatment related complications, or study-related complications
4. Patient status at follow-up: To further characterize patient outcomes following discharge after successful chest-tube removal, additional data will be collected regarding patient status at their follow-up visit and characterized based on five scenarios:
   1. Follow-up: resolved infection: Upon clinician follow-up there is no further evidence of residual infection clinically and on imaging and no further need for evaluation or treatment for infection resolution.
   2. Follow-up: surveillance: Upon clinician outpatient follow-up there is clinical concern for potential continued or residual infection despite initial chest tube drainage, requiring further clinical evaluation and potentially further procedures for resolution.
   3. Follow-up: ongoing infection: Upon clinical outpatient follow-up there is clear imaging or clinical signs of ongoing pleural infection despite initial chest tube drainage, actively requiring further management.
   4. Patient lost to follow-up: Following hospital discharge, the patient has been unable to be contacted or not presenting to outpatient appointments.
   5. Patient is deceased: Following hospital discharge, the patient is deceased. If available, cause of death will be documented.

# **Multistate Transition Model:**

Our data involves 3 processes: 1) whether patients received tPA/DNase therapy or not, 2) whether patient’s chest tube was deemed clogged vs patent, after appropriate steps taken to unclog a tube, and 3) whether the chest tube ± tPA/DNase was considered a treatment failure, requiring additional invasive procedures. Patients could experience a clogged tube but continue to have ongoing treatment in the form of antibiotics, ongoing drainage, and possible tPA/DNase (as opposed to treatment failure). Patients could transition between states of patent, vs clogged and tPA/DNase free vs tPA/DNase therapy, but if a patient was considered a treatment failure, this state was defined as an absorbing state/terminal state.

Considering these three processes yielded twelve possible states, or categories (see above): 1) patent chest tube with treatment successful, no tPA/DNase (in the past 24 hours), 2) clogged chest tube with treatment successful, no tPA/DNase, 3) patent chest tube with tPA/DNase, treatment successful, 4) clogged chest tube with treatment successful, with tPA/DNase, 5) patent chest tube with ongoing treatment, no tPA/DNase 6) patent chest tube with tPA/DNase with ongoing treatment, 7) clogged chest tube with ongoing treatment, no tPA/DNase 8) clogged chest tube with tPA/DNase with ongoing treatment, 9) patent chest tube with failed treatment, no tPA/DNase 10) patent chest tube with tPA/DNase with failed treatment, 11) clogged chest tube with tPA/DNase with failed treatment and 12) clogged chest tube with failed treatment.

We define four ordinal levels of status based on each category and each patient’s status will be assessed daily from randomization to treatment success, or failure. At any one point, a patient can be within one state, or category, and reaching any absorbing group, defined as treatment success, or treatment failure (groups 1 or groups 4) results in a terminal state with no further transitions possible.

Definition of States**:***Absorbing state* a terminal state, in which the probability of leaving that state is zero. 

*Treatment Successful* notes inpatient effusion management is complete. Chest tube can be removed, and patient could be discharged from a pleural infection standpoint. This does not include those discharged with a chest tube in place for ongoing management.

*Treatment failed* notes the patient needs an additional procedure for management of their pleural space (e.g., additional chest tube, surgery, etc.).

*Patency of tube* is assessed by the presence of tidaling and/or evidence of ongoing drainage output (*clogged vs. patent)*.

# **Statistical Considerations**

*Objective and sample size justification*

This is an open label, randomized, multicenter study of the application of flushing for the treatment of pleural space infection among hospitalized patients requiring chest tube drainage. Patients will be enrolled and randomized with a 1:1 ratio to two treatment groups: (1) 20 mL of sterile saline catheter flushing every 6±2 hours with the application of continuous -20 cm H2O suction (2) as needed saline flushing for blockage with the application of continuous -20 cm H2O suction. After enrollment, daily drain patency and criteria for drain removal will be measured and documented. The primary objective of this study is to evaluate the impact of regular chest tube drain flushing on the length of time to catheter removal for patients admitted with pleural space infection who require thoracostomy for their management. Chest tube removal due to chest tube dislodgement or malposition, or “kinking”, and if the patient is deceased with a chest tube in situ, are considered competing risks and will be analyzed as such.

The secondary objective of this study is to evaluate the impact of regular chest drain flushing on the length of hospitalization for patients, radiographic improvement on chest imaging, the need for additional pleural interventions, and associated complications of chest tube management.

Previous studies have shown a varying number of days to chest tube removal based on etiology, with a mean of 7.6 days (SD: 1.53) in no regular flush populations in a study of 98 patients with infected pleural space (Cafarotti). There is limited data with only one small study reporting median days to chest tube removal (5 days (3-7) in a study of 36 patients with no-flush strategy) (Horsley). Assuming a non-normal distribution, with data skewed to the right, we estimated a median of 6 days to chest tube removal. Assuming a difference of 1 day (24 hours), earlier chest tube removal in the intervention (6 days in the SOC group and 5 days in the intervention group) is a clinical meaningful difference to detect. The sample size is calculated from a two-sided Wilcoxon Rank-Sum test at a 0.050 significance level with a standard deviation of 1.5. Group sample sizes of 38 in each arm achieve 80% power to detect a median difference of 1. The sample size estimation is based on a 2000 Monte Carlo simulation assuming samples from a range of distribution such as Gamma, Lognormal, and Gumbel distributions. Assuming a 10% withdrawal rate and 15% intrapleural enzyme therapy failure (which can prevent reaching the primary endpoint), we plan to enroll 96 Participants (with randomization performed in a 1:1 ratio). To avoid an unbalance in randomization among groups, randomization will be stratified by intrapleural enzyme therapy use.

The main analysis for the outcome will be performed according to the intention-to-treat principle; all the participants with an observed outcome will be included in the analysis according to their assigned trial group.

*Analysis plans*

Continuous variables will be summarized using the mean (SD) or median (range), as appropriate. Frequencies and percentages will be used to summarize categorical variables. The student t-test or the Wilcoxon test will be used to compare continuous variables between two different groups. The chi-square test or Fisher’s exact test will be applied to assess the association between two categorical variables such as complications by treatment.

The Wilcoxon rank sum test will be used to compare time from randomization (within 24 hours of chest tube placement) until time to chest tube removal between two arms among patients who achieved successful chest tube removal.

For the primary endpoint, if data is normally distributed, data will be analyzed via students t-tests comparing time from randomization until time to chest drain removal between the two arms, among patients who received successful chest drain removal. For non-normally distributed data, the Wilcoxon rank sum test will be used for this comparison. If a chest drain’s removal was due to a competing risk (chest drain dislodgement, malposition or “kinking”, patient died with chest drain in situ), a competing risk time-to-event regression model will be applied.

In addition to the primary endpoint of interest, we are interested in the comparison of the time to the failure of therapy/drainage between two arms. For the time to the failure of therapy/drainage analysis, Cox regression with the adjustment of covariates of interest will be used in all enrolled subjects, including patients who reached successful chest tube removal and those with failure of therapy/drainage and need for additional interventions. The cumulative failure of therapy/drainage curve will be calculated from the Kaplan-Meier method and compared using the log-rank test. For the exploratory study, we will apply Markov ordinal longitudinal proportional odds state transition model to analyze our interventions into impact on the process of chest tube drainage to successful outcome or failure. This model will provide further characterization of intervention and control arms with regards to state of chest drain and state of patient among all populations, including those with chest drain failure, to characterize differences among these groups.

# **Informed Consent/Authorization**

Patients will have consented to participate in this study.

The informed consent will be obtained by the RELIEF study coordinator or one of the investigators of the study with a clear description of the purpose and procedures of the study, the implications for enrolled patients in terms of clinical care whether they decide to participate in the study or not, the potential risks and benefits of the study. It will be made clear to participants that they will be at liberty to withdraw from the study at any point or decline participation outright without influencing their level of clinical care. Oral and written consent will be obtained with the signed informed consent archived in the research department of the Division of Pulmonary & Critical Care at Vanderbilt University. The patient will be furnished with a copy of the signed study documents for his or her personal records.

# **Data Confidentiality and Monitoring**

Study data will be collected and managed using REDCap (Research Electronic Data Capture) electronic data capture tools hosted at Vanderbilt University. REDCap (www.project-redcap.org) is a secure, web-based application with controlled access designed to support data capture for research studies, providing: 1) an intuitive interface for validated data entry; 2) audit trails for tracking data manipulation and export procedures; 3) automated export procedures for seamless downloads to common statistical packages; and 4) procedures for importing data from external sources (Harris). In the case of multi-center studies, REDCap uses Data Access Groups (DAGs) to ensure that personnel at each institution are blinded to the data from other institutions.

Vanderbilt will have access to the data captured at the research sites and will use the data combined from all databases for complete analyses.

Following publication study data will be archived in REDCap. Since study data may be useful for future research studies performed under separate IRB-approved protocols, study data will be archived indefinitely in REDCap. Since REDCap is a secure electronic database with controlled access, and because patient identifiers may be needed to link study data to data from other sources under future IRB-approved protocols, patient identifying information will be retained in the archived database.

# **Safety**

We do not expect additional safety concerns from this protocol over those incurred during conventional placement and maintenance of chest tubes. These risks, inherent to the chest tube procedure itself, are discussed as part of the clinical informed consent and include bleeding, infection, pain, and pneumothorax. No interventions outside the standard of care of performed in this study.

*Adverse event (AE)*

An adverse event (AE) is any untoward medical occurrence in a patient administered a pharmaceutical product, which does not necessarily have a causal relationship with the treatment. This study is expected to have minimal to no adverse effects.

Treatment-related adverse events will be collected for this study. Therefore, data, including adverse events, will be collected from patients pre- or post-treatment. Only adverse events related to research procedures (the consent process, pain assessment, HIPAA compliance, etc.) will be collected.

Serious adverse events will be recorded as part of the study.

*Serious adverse event (SAE)*

This study will not have any serious adverse effects. A serious adverse event (SAE) is an undesirable sign, symptom, or medical condition which:

- is fatal or life-threatening.
- requires or prolongs inpatient hospitalization.
- results in persistent or significant disability/incapacity.
- jeopardizes the participant and requires medical or surgical intervention to prevent one of the outcomes listed above.

Events not considered to be serious adverse events are hospitalizations for:

- Routine treatment or monitoring of the studied indication, not associated with any deterioration in condition, or for elective procedures.
- Elective or pre-planned treatment for a pre-existing condition that did not worsen.
- Emergency outpatient treatment for an event not fulfilling the serious criteria outlined above cannot resulting in inpatient admission.
- Respite care.

General Instructions for Reporting Serious Adverse Events

The Institutional Review Board will be notified of all SAEs, within 7 business days after the treating institution becomes aware of the event. Only SAEs related to research procedures will be reported to the IRB.

*Benefits*There is no guarantee that the subjects will receive any benefit from this study.

# **Finances**

*Payment and Remuneration*

Subjects will not be paid to participate in the study.

*Costs*

There will be no additional costs to subjects for participating in this study. Subjects and/or their insurance companies will be responsible for part of the care provided as this service is part of the standard of care they would receive for their condition.

*Budget*

Funding provided by Cook Medical Inc.

# **References**

Najafi S, Sandrock C. Hospitalized Patients with Acute Pneumonia. Hosp Med Clin. 2017 Oct;6(4):456-469. doi: 10.1016/j.ehmc.2017.05.012. Epub 2017 Sep 15. PMID: 32288999; PMCID: PMC7104154.

Peters, Z. J., Ashman, J. J., Schwartzman, A., & DeFrances, C. J. (2022). National Hospital Care Survey Demonstration Projects: Examination of Inpatient Hospitalization and Risk of Mortality Among Patients Diagnosed With Pneumonia. National health statistics reports, (167), 1–9.

Sorino, C., Mondoni, M., Lococo, F., Marchetti, G., & Feller-Kopman, D. (2022). Optimizing the management of complicated pleural effusion: From intrapleural agents to surgery. Respiratory medicine, 191, 106706. <https://doi.org/10.1016/j.rmed.2021.106706>

Shen, K. R., Bribriesco, A., Crabtree, T., Denlinger, C., Eby, J., Eiken, P., Jones, D. R., Keshavjee, S., Maldonado, F., Paul, S., & Kozower, B. (2017). The American Association for Thoracic Surgery consensus guidelines for the management of empyema. The Journal of thoracic and cardiovascular surgery, 153(6), e129–e146. <https://doi.org/10.1016/j.jtcvs.2017.01.030>

Light R. W. (2006). Parapneumonic effusions and empyema. Proceedings of the American Thoracic Society, 3(1), 75–80. <https://doi.org/10.1513/pats.200510-113JH>

Davies, H. E., Davies, R. J., Davies, C. W., & BTS Pleural Disease Guideline Group (2010). Management of pleural infection in adults: British Thoracic Society Pleural Disease Guideline 2010. Thorax, 65 Suppl 2, ii41–ii53. <https://doi.org/10.1136/thx.2010.137000>

Maskell, N. A., Davies, C. W., Nunn, A. J., Hedley, E. L., Gleeson, F. V., Miller, R., Gabe, R., Rees, G. L., Peto, T. E., Woodhead, M. A., Lane, D. J., Darbyshire, J. H., Davies, R. J., & First Multicenter Intrapleural Sepsis Trial (MIST1) Group (2005). U.K. Controlled trial of intrapleural streptokinase for pleural infection. The New England journal of medicine, 352(9), 865–874. <https://doi.org/10.1056/NEJMoa042473>

Cafarotti, S., Dall'Armi, V., Cusumano, G., Margaritora, S., Meacci, E., Lococo, F., Vita, M. L., Porziella, V., Bonassi, S., Cesario, A., & Granone, P. (2011). Small-bore wire-guided chest drains: safety, tolerability, and effectiveness in pneumothorax, malignant effusions, and pleural empyema. The Journal of thoracic and cardiovascular surgery, 141(3), 683–687. <https://doi.org/10.1016/j.jtcvs.2010.08.044>

Davies, H. E., Merchant, S., & McGown, A. (2008). A study of the complications of small bore 'Seldinger' intercostal chest drains. Respirology (Carlton, Vic.), 13(4), 603–607. <https://doi.org/10.1111/j.1440-1843.2008.01296.x>

Porcel, J. M., Valencia, H., & Bielsa, S. (2017). Manual Intrapleural Saline Flushing Plus Urokinase: A Potentially Useful Therapy for Complicated Parapneumonic Effusions and Empyemas. Lung, 195(1), 135–138. <https://doi.org/10.1007/s00408-016-9964-2>

Horsley, A., Jones, L., White, J., & Henry, M. (2006). Efficacy and complications of small-bore, wire-guided chest drains. Chest, 130(6), 1857–1863. <https://doi.org/10.1378/chest.130.6.1857>

Dorman S, Jolley C, Abernethy A, et al. Researching breathlessness in palliative care: consensus statement of the National Cancer Research Institute Palliative Care Breathlessness Subgroup. Palliative medicine 2009;23:213-27.

Ries AL. Minimally clinically important difference for the UCSD Shortness of Breath Questionnaire, Borg Scale, and Visual Analog Scale. Copd 2005;2:105-10.
